# Magnetization Transfer Ratio of Peripheral Nerve and Skeletal Muscle: Correlation with Demographic Variables in Healthy Volunteers

**DOI:** 10.1007/s00062-021-01067-5

**Published:** 2021-08-10

**Authors:** Olivia Fösleitner, Véronique Schwehr, Tim Godel, Fabian Preisner, Philipp Bäumer, Sabine Heiland, Martin Bendszus, Moritz Kronlage

**Affiliations:** 1 Department of Neuroradiology, Heidelberg University Hospital, Im Neuenheimer Feld 400, 69120 Heidelberg, Germany; 2 diadia.log, Altötting Center for Radiology, Altötting, Germany

**Keywords:** Magnetic resonance imaging, Peripheral nervous system, Demography, Reference values

## Abstract

**Purpose:**

To assess the correlation of peripheral nerve and skeletal muscle magnetization transfer ratio (MTR) with demographic variables.

**Methods:**

In this study 59 healthy adults evenly distributed across 6 decades (mean age 50.5 years ±17.1, 29 women) underwent magnetization transfer imaging and high-resolution T2-weighted imaging of the sciatic nerve at 3 T. Mean sciatic nerve MTR as well as MTR of biceps femoris and vastus lateralis muscles were calculated based on manual segmentation on six representative slices. Correlations of MTR with age, body height, body weight, and body mass index (BMI) were expressed by Pearson coefficients. Best predictors for nerve and muscle MTR were determined using a multiple linear regression model with forward variable selection and fivefold cross-validation.

**Results:**

Sciatic nerve MTR showed significant negative correlations with age (r = −0.47, *p* < 0.001), BMI (r = −0.44, *p* < 0.001), and body weight (r = −0.36, *p* = 0.006) but not with body height (*p* = 0.55). The multiple linear regression model determined age and BMI as best predictors for nerve MTR (R^2^ = 0.40). The MTR values were different between nerve and muscle tissue (*p* < 0.0001), but similar between muscles. Muscle MTR was associated with BMI (r = −0.46, *p* < 0.001 and r = −0.40, *p* = 0.002) and body weight (r = −0.36, *p* = 0.005 and r = −0.28, *p* = 0.035). The BMI was selected as best predictor for mean muscle MTR in the multiple linear regression model (R^2^ = 0.26).

**Conclusion:**

Peripheral nerve MTR decreases with higher age and BMI. Studies that assess peripheral nerve MTR should consider age and BMI effects. Skeletal muscle MTR is primarily associated with BMI but overall less dependent on demographic variables.

**Supplementary Information:**

The online version of this article (10.1007/s00062-021-01067-5) contains supplementary material, which is available to authorized users.

## Introduction

Magnetic resonance neurography (MRN) enables imaging of peripheral nerve morphology and pathology in vivo. Fat-saturated T2-weighted sequences are increasingly used in clinical routine settings and reliably detect neuropathy on a fascicular level, e.g. in trauma and inflammation [1, 2]. While T2 hyperintensity is a sensitive biomarker of neuropathy, it is unspecific and found in various etiologies [1]. Due to their very short T2 relaxation times (< 1 ms), protons bound to macromolecules, such as myelin, are practically invisible in standard MRN sequences with usual echo times around 40–60 ms [3]. As such, T2 hyperintensity does not differentiate between a mere increase in free water content (edema) and a decay of structural components (e.g. demyelination) [4].

Magnetization transfer imaging (MTI) could provide one way to overcome this limitation and thus enable discrimination of nerve tissue pathologies more specifically:

While the short T2 relaxation time of protons bound to macromolecules (< 1 ms) impedes their direct visualization, resonance occurs in a much broader bandwidth off the Larmor frequency compared to free-water protons. The MTI uses such an off-resonance radio frequency pulse that selectively saturates the pool of protons bound to macromolecules while not significantly saturating the pool of free-water protons due to their smaller bandwidth of magnetic resonance [5]. According to the two-pool model, magnetization is then transferred from the pool of protons bound to macromolecules to the pool of protons in free water [3]. The magnetization transfer ratio (MTR) is defined as the relative signal difference between two sequences, one with and one without the off-resonance pulse, with otherwise identical technical parameters. The MTR was shown to be a sensitive marker of demyelination in the central nervous system in multiple sclerosis [6, 7]. Similarly, MTI offers a novel contrast in peripheral nerve imaging and MTR was assessed as a novel quantitative peripheral nerve imaging biomarker in first studies of amyloidosis [8] and Charcot-Marie-Tooth diseases [9].

Age and body constitution are known influencing factors of nerve morphology [10, 11]. Specifically, correlations with demographic variables were found for the quantitative MR neurography biomarkers of nerve cross-sectional area [12], diffusion tensor imaging (DTI) metrics [13], and the T2-relaxometry-based proton spin density [12].

To our knowledge, a systematic assessment of possible correlations between peripheral nerve MTR and demographic variables in a larger cohort of healthy volunteers has not been conducted yet, while results of a first exploratory study with a small cohort of 10 younger and 5 older healthy subjects indicated that MTR might decrease with age [14].

Similar to peripheral nerve MTR, skeletal muscle MTR is being evaluated as an imaging biomarker of muscle pathology, such as neurogenic denervation and primary myopathy. While healthy muscle tissue is rich in complex macromolecules, muscle atrophy and fatty degeneration are accompanied by a decline in the complexity of macromolecular structure and thus measurably lower MTR values [9, 15, 16]. Data on possible correlations of skeletal muscle MTR with demographic variables are still scarce and in part contradictory [17, 18].

Demographic effects are crucial to consider when investigating disease-specific MTR changes. The aim of this study was to assess the correlation of peripheral nerve MTR and skeletal muscle MTR with demographic variables in a larger cohort of healthy volunteers.

## Material and Methods

### Subjects

This study was approved by the institutional ethics committee and written informed consent was obtained from all participants. The study was conducted in accordance with the Declaration of Helsinki.

A total of 60 healthy volunteers (30 female, 30 male) evenly distributed across the decades between 20 years and 80 years old were prospectively recruited by public announcement from January 2016 to October 2016, as described before [12]. Exclusion criteria were any known neurologic or systemic diseases and general contraindications against an MRI examination as well as insufficient image quality.

For each participant age, body height and weight, body mass index (BMI), arterial hypertension and smoking status were registered based on self-reported assessment. Hypertension was defined as drug-treated hypertension, smoking status was positive if participants had a history of at least 2 pack years.

### Magnetic Resonance Neurography

All subjects were examined in a 3.0 T MR scanner (Magnetom Tim Trio; Siemens Healthineers, Erlangen, Germany) in supine position. A 15-channel transmit-receive knee coil (Siemens Healthineers) was used. The MTI was performed by applying two axial proton density-weighted, gradient echo sequences with and without an off-resonance saturation pulse with otherwise identical parameters and identical positioning at the distal thigh: repetition time: 46 ms; echo times: 4.92 ms, 12.3 ms, 19.68 ms and 27.06 ms; field of view: 160 × 160 mm^2^; matrix size: 128 × 128; bandwidth: 369 Hz/Px; 24 slices; slice thickness: 4.0 mm; slice gap: 0.8 mm; number of excitations: 1; flip angle: α = 7°; Gaussian envelop, duration = 9984 μs, frequency off-set = 1200 Hz; acquisition time: 2 min 26 s. An adaptive inline image filter was applied to reduce B1 field inhomogeneities (Siemens Healthineers). For precise anatomical nerve segmentation, an axial T2-weighted turbo spin echo sequence was additionally acquired as previously described [12]: repetition time: 8150 ms; echo time: 54 ms; field of view: 160 × 160 mm^2^; matrix size: 512 × 333; bandwidth: 181 Hz/Px; 41 slices; slice thickness: 3.5 mm; slice gap: 0.35 mm; number of excitations: 2; flip angle: α = 150°; acquisition time: 4 min 22 s.

### Image Postprocessing

**Fig. 1:**
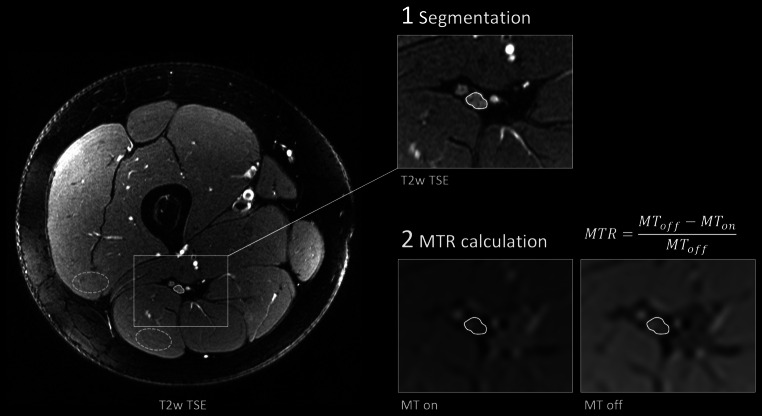
Representative images of nerve segmentation. The tibial portion of the sciatic nerve was first delineated on T2-weighted (T2w) images providing excellent anatomical contrast. Hereafter, the region of interest was transferred onto magnetization transfer (MT) images with (MT on) and without (MT off) an off-resonance saturation pulse and manually corrected for distortion and chemical shift artifacts. Finally, the MT ratio (MTR) was calculated. Representative regions of interest for muscle MTR calculation are illustrated with *dashed lines*

Image analysis was conducted in OsiriX Version 11 (Pixmeo Sàrl, Bernex, Switzerland) by O.F. with more than 4 years of experience in neuromuscular radiology. First, visual assessment of image quality was performed. As illustrated in Fig. 1, the tibial portion of the sciatic nerve was then segmented in six consecutive slices of the T2-weighted sequence using a free-hand region of interest (ROI). To prevent inclusion of paraneurial fat that separates the tibial and peroneal portion of the sciatic nerve to a variable degree, analysis was restricted to the tibial portion similar to studies of other quantitative MR neurography biomarkers [12, 13, 19]. In order to avoid edge-related artifacts, six representative slices in the center of the stack were used. Only MT images at the shortest echo time (4.92 ms) were used for analysis. The ROIs were transferred to the MTR sequence and manual correction of distortion and chemical shift artifacts was conducted in the image without off-resonance saturation pulse. The MTR was calculated according to the following equation:

$$MTR=\frac{MT_{\mathrm{off}}-MT_{on}}{MT_{\mathrm{off}}}$$

Where MT_off_ and MT_on_ are the mean nerve signal intensities with and without off-resonance saturation pulses. For further calculations, mean values from the six consecutive slices were used.

To assess muscle MTR, oval-shaped ROIs were placed in homogeneous areas of the biceps femoris and vastus lateralis muscles at corresponding slices and muscle MTR was calculated analogously to the nerve. Exemplary MTR maps were calculated with a custom-written Python script and FIJI software (version 2.1.0 [20]) for illustrative purposes.

### Statistical Analysis

Statistical analysis was performed in SPSS Version 26 (IBM, Armonk, NY, USA) and Prism Version 8 (GraphPad Software, La Jolla, CA, USA). Pearson’s correlation coefficient was used to describe correlations among demographic variables, and between demographic metrics and MTR values. Independent t‑test was performed to assess sex-related MTR differences, and non-parametric Mann-Whitney test for hypertension and smoking status. One-way analysis of variance was used to compare MTR between tibial nerve, biceps femoris and vastus lateralis muscles with subsequent Tukey’s test for multiple comparisons. A multiple linear regression model with forward variable selection (entry threshold *P* ≤ 0.05) for nerve MTR and muscle MTR was performed to determine the best predictors among demographic variables. The (predictive) R^2^ value was determined with fivefold cross-validation, i.e. the dataset was randomly split into five portions and linear regression was then calculated for each combination of subsets by leaving one out. Significance level was set at *P* ≤ 0.05. Results are given as mean values ± standard deviation unless indicated otherwise.

## Results

Demographic details are outlined in Table 1. There was no significant association between age and body height (r = −0.07, *p* = 0.59), age and body weight (r = 0.001, *p* = 0.99) or age and BMI (r = 0.07, *p* = 0.59). Body height, weight, and BMI but not age differed significantly between male and female participants. One woman was excluded due to insufficient image quality. Thus, the data of 59 participants were finally analyzed in this study.

**Table 1:** Demographic characteristics of study participants. Values are mean ± standard deviation

	Total (*n* = 59)
Age (years)	50.5 ± 17.1
Height (cm)	174.4 ± 9.6
Weight (kg)	75.8 ± 16.4
BMI (kg/m^2^)	24.7 ± 3.9

*BMI* body mass index

**Fig. 2:**
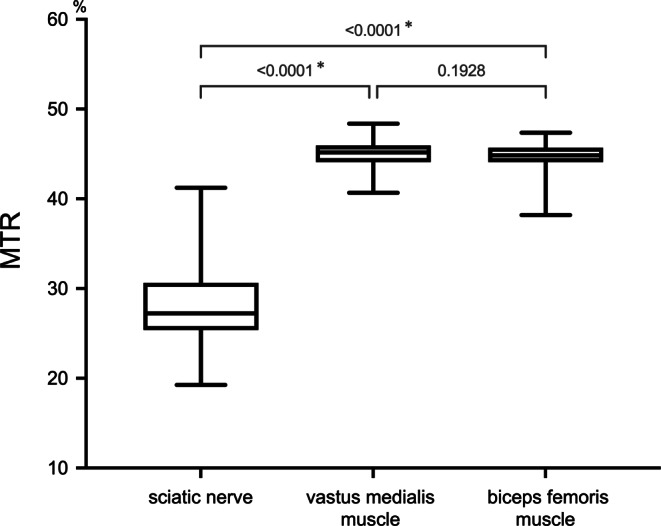
Comparison of nerve and muscle magnetization transfer ratio (MTR) values. Median value is indicated by the horizontal line within the box-and-whisker plot. The box length shows the interquartile range, and whiskers represent the range of data. Sciatic nerve MTR significantly differs from MTR of vastus medialis and biceps femoris muscles but not between muscles. * indicates significance (*p* ≤ 0.05)

Mean MTR of the sciatic nerve (28.0 ± 4.7%) was significantly lower than MTR of the biceps femoris (44.5 ± 1.8%, *p* < 0.0001) and vastus medialis muscles (44.9 ± 1.6% *p* < 0.0001), as presented in Fig. 2. The MTR values between both muscles did not differ significantly (*p* = 0.19). There was no significant difference of nerve or muscle MTR between male and female participants. Likewise, both muscle and peripheral nerve MTR of smokers (7/59 participants) and non-smokers as well as of hypertonic (6/59 participants) and normotonic subjects were not significantly different (Supplementary Table).

**Fig. 3:**
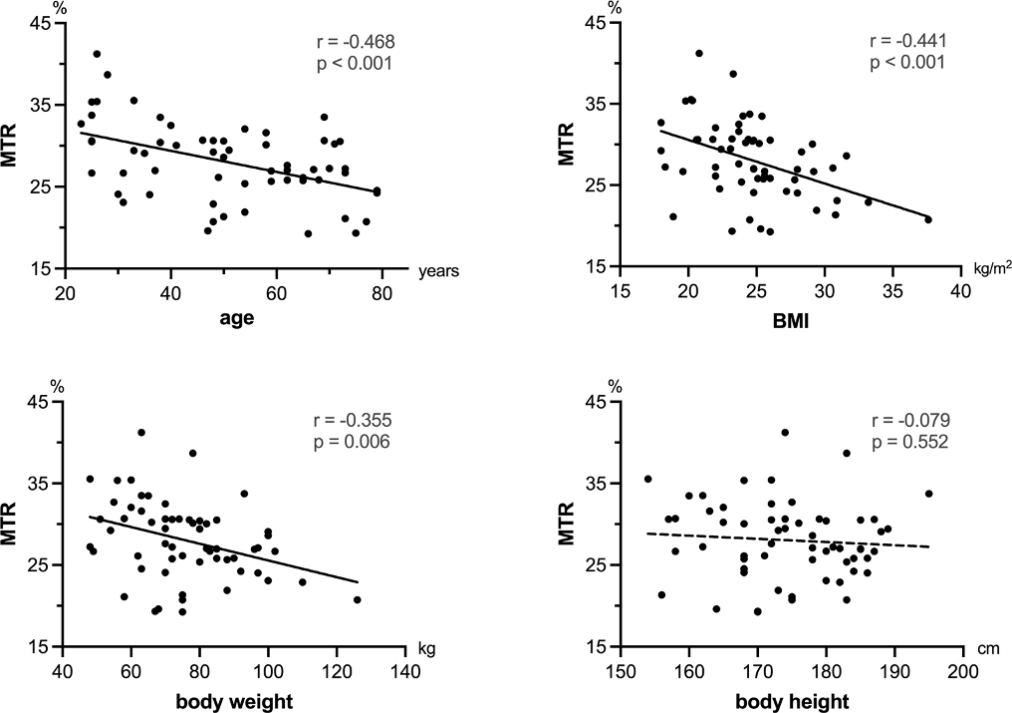
Correlation of sciatic nerve magnetization transfer ratio (MTR) and the demographic variables of age, BMI, body weight and height. Nerve MTR across all participants was negatively associated with age, BMI and body weight, but not with body height. *r* Pearson coefficient

Correlations of demographic variables with peripheral nerve MTR are shown in Fig. 3. Among the assessed variables, the strongest correlation was found for age and peripheral nerve MTR (r = −0.47, *p* < 0.001). Likewise, BMI and weight were associated with nerve MTR (MTR/BMI: r = −0.44, *p* < 0.001; MTR/weight: r = −0.36, *p* = 0.006). No significant correlation was found for nerve MTR and body height (r = −0.08, *p* = 0.55).

A multiple linear regression model with nerve MTR as the dependent variable was calculated. Forward variable selection among the demographic variables of age, body height, body weight, and BMI included age and BMI as best predictors in the model, achieving an R^2^ of 0.40 (*p* < 0.001). Using fivefold cross-validation this model yielded a mean R^2^ of 0.43.

The model can be described by the formula:

$$MTR\left[\% \right]=46.655-0.127x\frac{age}{\textit{years}}-0.493x\frac{\mathrm{BMI}m^{2}}{kg}.$$

**Fig. 4:**
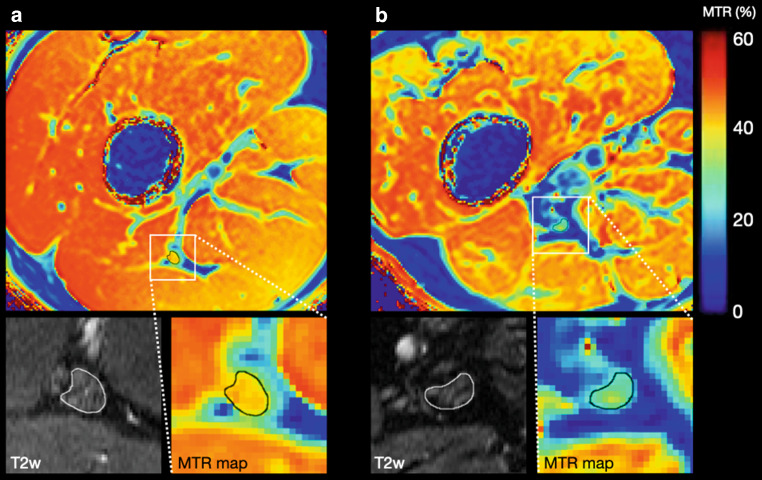
Representative magnetization transfer ratio (MTR) pseudo-colorized (%) maps of a 28-year-old (**a**) and a 77-year-old (**b**) healthy male volunteer. Boxes below show the zoomed sciatic nerve in T2-weighted (T2w) images and MTR maps. Note the decrease in MTR in the older subject compared to the younger individual

Standardized beta coefficients were −0.46 for age (95% CI −0.67, −0.25) and −0.40 for BMI (95% CI −0.61, −0.20). The age-dependent effect is illustrated with MTR maps of two representative participants in Fig. 4.

**Fig. 5:**
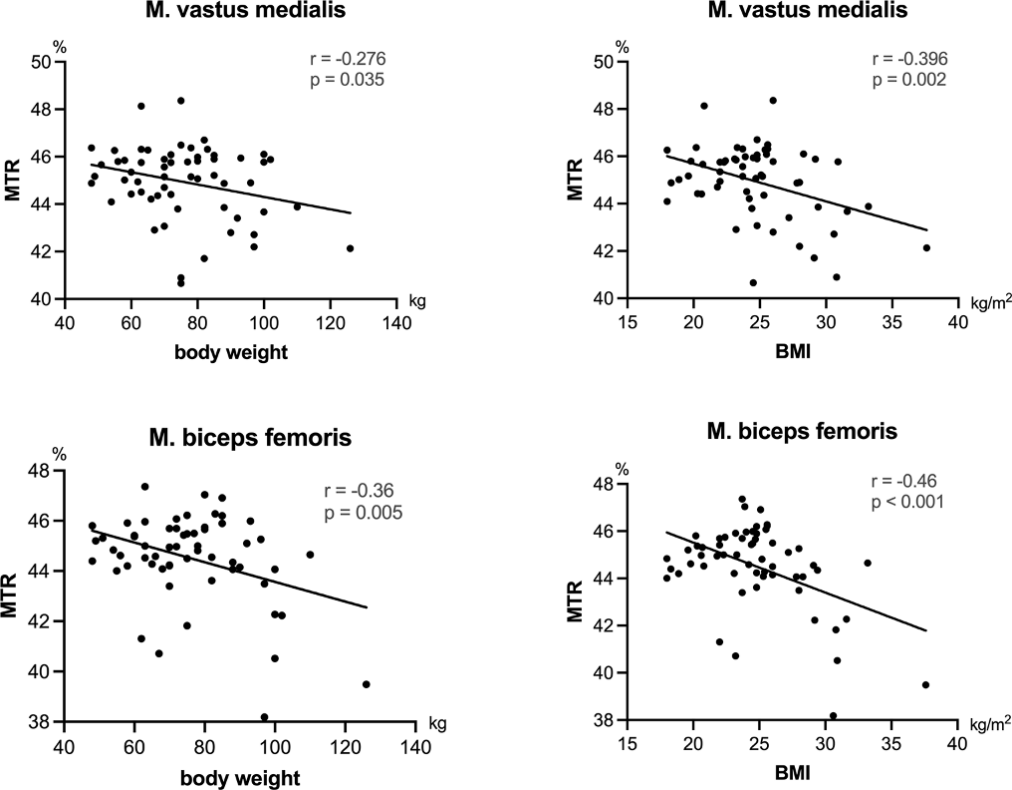
Correlation of biceps femoris and vastus medialis magnetization transfer ratio (MTR) with selected anthropomorphic measures. Body weight and BMI showed a negative correlation with both the vastus medialis and the biceps femoris muscle. *r* Pearson coefficient

The MTR of both biceps femoris and vastus medialis muscles correlated with body weight (r = −0.36, *p* = 0.005; r = −0.28, *p* = 0.04) and BMI (r = −0.46, *p* < 0.001; r = −0.40, *p* = 0.002) but not with age or body height (Fig. 5). A multiple linear regression model was calculated for skeletal muscle MTR (mean MTR of biceps femoris and vastus medialis muscles). By forward variable selection, only BMI was included as a predictor achieving an R^2^ value of 0.26 (*p* < 0.001) and cross-validated mean R^2^ of 0.28. The standardized beta coefficient was −0.51 for BMI (95% CI −0.74, −0.28).

## Discussion

Magnetization transfer imaging offers a promising new contrast in magnetic resonance neurography, indirectly measuring the macromolecular composition of nerve and muscle tissue. This study represents the first systematic correlation analysis of peripheral nerve and muscle magnetization transfer ratio with demographic parameters in a larger collective of 59 healthy volunteers. Age and BMI were found as best predictors of nerve MTR, while muscle MTR was mainly dependent on BMI. The reported effects are important to consider when investigating disease-specific MTR changes.

Among the assessed demographic variables, the most important determinant of peripheral nerve MTR was age. We report a negative association of age and peripheral nerve MTR which is in line with findings of a first study by Kollmer et al. [14] who reported a lower mean value of nerve MTR in a small group of older participants (*n* = 5) compared to a group of younger subjects (*n* = 10). Similarly, MTR of gray and white matter in the brain was found to decrease with higher age [21]. Age-dependency of peripheral nerve metrics was also shown for DTI [13] and electrophysiology [11]. On a cellular level, increasing age is related to multiple changes in nerve morphology and function, including loss of nerve fibers, alterations in the myelin sheath, and axonal atrophy [10, 11]. Further studies are needed to investigate which of these changes exactly lead to the decline in MTR.

Moreover, we report inverse correlations between nerve MTR and BMI as well as nerve MTR and body weight. To our knowledge, these associations have not been identified before. Body constitution parameters have been reported to correlate with other morphological and functional MR neurography parameters, such as cross-sectional area [12], proton spin density [12], and DTI metrics [13, 22]. Likewise, associations of nerve morphology (cross-sectional area) with BMI and weight have also been described in ultrasound studies [23–25]. Intuitively, higher body weight and BMI are accompanied by increased perineurial fat, which might modify peripheral nerve MTR by partial volume effects; however, the interaction between body weight and peripheral nerve metabolism may be more complex. Animal studies indicate that adipose tissue mediates neurogenic inflammation and ultimately structural changes [26]. Obesity is closely related to type 2 diabetes which itself is a known risk factor for peripheral neuropathy [27], thus the histologic mechanism behind the association of BMI, body weight and MTR is difficult to discern.

Since demographic variables such as BMI and body weight may cross-correlate with each other, we calculated a multiple linear regression model with forward variable selection to determine which demographic variables should primarily be considered as determinants of peripheral nerve MTR. Including age and BMI in the model, it succeeded in explaining 40% of the observed variance of peripheral nerve MTR (R^2^ = 0.40) and it did not significantly improve with inclusion of further demographic variables. Therefore, we recommend to primarily control for age and BMI in further studies assessing peripheral nerve MTR.

Similar to peripheral nerve MTR, skeletal muscle MTR is discussed as an emerging biomarker of muscle pathology and has been assessed in Charcot-Marie-Tooth disease, chronic inflammatory demyelinating polyneuropathy, and inclusion body myositis [9, 15, 16].

We found muscle MTR to be associated with body weight and BMI but not with age, body height or sex. Since weight and BMI intercorrelate with each other, we calculated a multiple linear regression model, which revealed BMI as the most important determinant; however, compared to nerve tissue overall variance of muscle MTR was smaller and the multiple linear regression model revealed that only 22% of variance of muscle MTR could be explained by variance in demographic parameters (R^2^ = 0.22).

Our findings of a negative correlation of thigh muscle MTR and BMI or weight are in line with a study by Morrow et al. [18]. One explanation could be that accumulation of adipose tissue appears as increased intramuscular and intracellular lipid deposition [28, 29]; however, determination of intramuscular fat fraction was beyond the scope of this study. As MTR is sensitive to both increases in fat and other structural myogenic changes, further investigations are required to determine the physiological correlate of this correlation. Age alone, on the contrary, did not lead to measurable differences in MTR in our cohort, similar to an earlier study in calf muscles [17]. In contrast to our own results, Morrow et al. found a significant negative correlation between age and MTR in both thigh and calf muscles [18].

Hypertension and smoking status were not significantly associated with MTR values of nerve or muscle in this study; however, due to the small number of hypertensive and smoking participants these findings have to be interpreted cautiously. Moreover, history of smoking, hypertension and demographic data were self-reported, which may influence the results. Another limitation of this study is that absolute MTR values are highly dependent on MRI sequence parameters, particularly on the design of the off-resonance saturation pulse [30]. Besides, other sequence parameters such as the application of fat-saturation are not standardized but influence the absolute MTR values [31]. Absolute MTR values can therefore currently not be considered as general reference values. Besides, B1 field inhomogeneities may not be fully corrected by the inline filter used. In order to implement MTI as a comparable technique, a standardized protocol would be desirable. Moreover, we restricted analysis to the sciatic nerve which is technically most suited for quantitative imaging due to its large cross-sectional area, its straight course and the good accessibility of the thigh with surface coils as standard knee coils.

In conclusion, we systematically analyzed the correlation of magnetization transfer ratio (MTR) as an emerging quantitative MR neurography biomarker with demographic variables in a cohort of 59 healthy participants. The most important finding was a negative association of sciatic nerve MTR with both age and BMI. Thigh muscle MTR was less dependent on demographic determinants. Among those, the most important determinant was BMI. Studies that further assess peripheral nerve MTR should therefore consider age and BMI effects, while BMI may be regarded a demographic determinant of skeletal muscle MTR.

## Supplementary Information

Supplemental table: Magnetization transfer ratio (MTR) in relation to sex, smoking status and arterial blood pressure. Values are median (minimum-maximum). *P* values are calculated with the independent t‑test (sex) or the Mann-Whitney test (smoking status, hypertension).

## Abbreviations

BMI: Body mass index
DTI: Diffusion tensor imaging
MRN: Magnetic resonance neurography
MTI: Magnetization transfer imaging
MTR: Magnetization transfer ratio
ROI: Region of interest
