# Supplementary material for: Magnetization Transfer Ratio of Peripheral Nerve and Skeletal Muscle: Correlation with Demographic Variables in Healthy Volunteers
Source: Clin Neuroradiol. 2021 Aug 10;32(2):557–64. doi: 10.1007/s00062-021-01067-5 (PMC9187530; doi:10.1007/s00062-021-01067-5)
Supplement: Supplementary file 1 — Supplemental table: Magnetization transfer ratio (MTR) in relation to sex, smoking status and arterial blood pressure. Values are median (minimum-maximum). P values are calculated with the independent t‑test (sex) or the Mann-Whitney test (smoking status, hypertension). [file 62_2021_1067_MOESM1_ESM.docx]

SUPPLEMENTARY

|  | |  | |  | | |  |  | |  | | |  |  | |  | | |  |
| --- | --- | --- | --- | --- | --- | --- | --- | --- | --- | --- | --- | --- | --- | --- | --- | --- | --- | --- | --- |
|  | Male | | Female | | *P* value | Non-smoker | | | Smoker | | *P* value | Normotension | | | Hypertension | | *P* value | | |
| Sciatic nerve MTR | 26.9  (20.7, 41.2) | | 30.0  (19.2, 35.6) | | 0.70 | 27.4  (19.2, 41.2) | | | 25.7  (20.7, 38.7) | | 0.78 | 28.6  (19.2, 41.2) | | | 26.3  (22.9, 33.5) | | 0.38 | | |
|  |  | |  | |  |  | | |  | |  |  | | |  | |  | | |
| Musculus vastus medialis MTR | 45.8  (40.7, 48.1) | | 45.0  (40.9, 48.4) | | 0.68 | 45.2  (40.7, 48.4) | | | 45.7  (42.1, 46.4) | | 0.91 | 45.2  (40.7, 48.4) | | | 44.2  (42.8, 46.7) | | 0.63 | | |
|  |  | |  | |  |  | | |  | |  |  | | |  | |  | | |
| Musculus biceps femoris MTR | 45.0  (38.2, 47.0) | | 44.6  (40.7, 47.4) | | 0.95 | 44.8  (38.2, 47.4) | | | 45.0  (39.5, 46.1) | | 0.89 | 44.8  (38.2, 47.4) | | | 44.9  (43.6, 46.3) | | 0.68 | | |
|  |  | |  | |  |  | | |  | |  |  | | |  | |  | | |
| **Supplemental table:** Magnetization transfer ratio (MTR) in relation to sex, smoking status and arterial blood pressure. Values are median (minimum-maximum). *P* values are calculated with the independent t-test (sex) or the Mann-Whitney test (smoking status, hypertension). | | | | | | | | | | | | | | | | | |  |  |
